# The Philosophy of Hygiène; or on Health in Its Relation to the Physical, Moral, and Political Régime of Modern Civilization

**Published:** 1842-10

**Authors:** 


ART. XII.

1. *Hygiène philosophique, ou de la Santé dans le Régime physique, moral, et politique de la Civilisation moderne.* Par J. J. VIREY, M.D. &c.—*Paris*, 1828. Tomes ii. 8vo, pp. 282, 312.

*The Philosophy of Hygiène ; or on Health in its relation to the physical, moral, and political Régime of modern Civilization.* By J. J. VIREY, M.D., &c.

2. *Essai d'Hygiène générale.* Par L. C. A. MOTARD, Docteur en Médecine de la Faculté de Paris.—*Paris*, 1841. Tomes ii. 8vo, pp. 496, 592.

*Essay on general Hygiène.* By Dr. L. C. A. MOTARD.

3. *The Principles of Population, and their Connexion with Human Happiness.* By ARCHIBALD ALISON, F.R.S.E., Advocate. 1840. Two vols. 8vo, pp. 572, 544.

STATE-MEDICINE, Medical Police, and Hygiène are terms which have been used synonymously to express the art or science which has for its object the application of the principles of medicine to securing the well-being and amelioration of society. We know that not a few persons connect with the term hygiène the ideas of diet and regimen, gymnastics, early-to-bed and early-to-rise doctrines, and the dull every-day matter relating to the health of valetudinarians. People are apt to think, too, that medical police implies nothing more than the seizure of stinking fish or unsound meat; or at most a fear-spreading contrivance termed a Board of Health, and brought into action when cholera rages. Hygiène is a science so little cultivated in England that we believe no treatise on it has yet appeared in our language, although we have literally cart-loads of valuable materials for such a treatise in our parliamentary reports.

Matters are managed better abroad, or at least in France and Germany; there governments and the press are alike interested in the subject. Indeed it is so hacknied, that scarcely a year passes in which we are not favoured with more than one philosophical, sentimental monograph on this captivating subject. Dr. Virey's publication is of this kind. Dr. Virey is very *gloriosous*, as all true Frenchmen are, very sentimental, and very philosophical; but his book has no practical value. He is a *fine* writer; that is, he has good and even noble ideas, but he conceals them amidst so much and such ridiculous magniloquence that it is painful to search for them. We fall upon an amusing specimen of his style at the close of the third book. Dr. Virey attempts a comparison between love and glory and their hygiènic effects on mankind under the form of a mythological fiction. At a grand levee Jupiter resolves to do a great action, and make man the first of living beings, as it is declared he shall be in "the book of destinies." To effect this he creates and sends to earth two genii, Eucle and Eros; the former taught by Minerva, the latter brought up in the bosom of Venus. When they get to earth, Eros gives "ardeur physique" to brutes as well as man; but Eucle, more dutiful, confines her gift—namely, the love of glory, to mankind. And so Dr. Virey defines man thus, *Homo, animal gloriæ.* Did Dr. Virey ever witness a cock-fight?

We have referred to Dr. Virey's work partly for the purpose of placing Dr. Motard's in a favorable point of view, and partly to exhibit the great progress the science of hygiène has made since 1828. The essay of the last-mentioned author commences by stating that the object of hygiène is, to satisfy the physical and moral wants of man in such a manner as may best advance his individual and social development; or, in other words, the health, ease, and welfare of the human race. The most natural method of considering the subject of his essay, Dr. Motard thinks would be, to determine the influence exercised upon mankind by the various governments, laws, and creeds, which have successively appeared in the world; by climates, and by the manners and customs of various nations, as regards diet, clothing, labour, &c.; as well as by all the physical circumstances which modify our organization so considerably. He thinks, however, that the present imperfect state of our knowledge, especially of ancient nations will not permit of this plan being adopted. In this opinion we scarcely agree with Dr. Motard, and we think when he peruses the work of Mr. Alison, which we shall presently notice, he will find that much of the inquiry he proposes has already been made, and in a manner equally philosophical and practical.

The plan adopted by Dr. Motard is the following. Referring to his definition he arranges the physical necessities of man under five heads. 1. The necessity of a habitation and a locality. 2. Of having food and drink. 3. Of taking care of the person. 4. Of labour. 5. Of preventing the attacks of epidemic diseases. He devotes no portion of his work to a consideration of the moral wants of man (and this we think is a defect); but in the various subdivisions he estimates the influence of each physical agent on the intellect and morals. His plan, as it is, is of a most comprehensive character, and embraces very numerous details. Into these we cannot at present enter, but as an example of his method we will cursorily notice the arrangement of his first book, in which he treats of "The necessity for a locality and an abode." The book is subdivided into three chapters, the first of which is devoted to a consideration of the atmosphere and soil, or of the earth and air; and the chapter contains three sections. In the first section is a general view of climates, comprising, *a*, their meteorology, *b*, their statistics. Under the head of meteorology, Dr. Motard notices the various climates of the globe in relation to their latitudes and seasons; height above the level of the sea; relations to the sun's rays, and to forests, mountains, deserts, rivers, lakes, &c.; the prevailing winds, hygrometric condition and purity of the atmosphere; intensity of light; magnetism and electricity. In the second section he considers—*a*, the special influence of climates on man as they vary in the particulars previously considered; and *b*, the general modifications climates exercise, in respect to mortality, formation of habits and customs, and their influence on the intellect and morals. In the third section the hygiènic rules to be deduced from the preceding considerations are stated; and first the best method of being acclimated, on changing from one climate to another, is considered, and the special rules applicable to individuals as they vary in age, sex, temperament, and state of health, and as the seasons vary. The second chapter of the first book treats of water in relation to climate, and particularly of stagnant water; this chapter also contains three sections. In the first, the subject is considered generally; the second is devoted to the special influence of marshes and of their varieties, and the third contains the hygienic rules proper to be observed, if we would obviate their bad effects, and render them useful. In the third chapter, Dr. Motard considers houses, cities, and villages, and follows strictly the triple subdivision into sections: first he notices them generally; then ascertains their influence on mankind under various modifying circumstances; and thirdly, states the hygiènic rules to be observed in reference to topography, hydrography, the nature and density of the population, &c.; and the best methods of constructing houses and public buildings. It may interest Mr. Chadwick to be informed that Dr. Motard allows from 866 to 1039 cubic feet to each patient in an hospital; and directs that the beds be at least a metre or about three feet three inches apart. The inspectors of prisons in England allow 1000 cubic feet to each prisoner.*

The plan of the succeeding four books is exactly the counterpart of that which we have detailed. The second book treats of food, condiments, and beverages; the third of articles of dress, baths, and gymnastics.

In the fourth the various occupations of mankind are reviewed as well as the subject of rest and sleep. Agriculture, war, commerce, navigation, and trades and professions as carried on in cities, are all noticed at length. The fifth and last book discusses epidemic and endemic diseases,—their nature and prophylaxis; and concludes, we do not see why, with a most meager chapter on the important subject of hereditary diseases. This and the preceding book are exceedingly interesting and deserve most careful perusal.

We think Dr. Motard has added materially to the value of his work by a statistical appendix, in addition to numerous statistical details scattered throughout the essay. This appendix contains thirty-three tables drawn up with special reference to the subjects discussed. In constructing these tables Dr. Motard, by making a very free use of English literature, has set a good and not unnecessary example to his countrymen. Tulloch, Marshall, Sir J. Clark, B. Hawkins, Macculloch, are some of the names quoted, somewhat disguised, it is true, according to French custom, but recognizable enough. Our periodicals are also laid under contribution; and Dr. Motard would no doubt have sunk a shaft into the mine of "blue books," if he had had an opportunity. Some of these tables are interesting: we do not remember to have seen the following:

"Table of the mean mortality in different degrees of latitude.

| | | | | |
|---|---|---|---|---|
| Within the tropics | 1 | death in | 25 | inhabitants. |
| From 20° to 40° | 1 | " | 34·5 | " |
| " 40° to 60° | 1 | " | 43·2 | " |
| " 60° to 70° | 1 | " | 50 | " |

(Table vi. p. 565. Tom. ii.)

Nor this:

" Ratio of mortality per cent. during the year 1807 in the hospitals of Paris, classed according to the occupations of the sick.

| Trade. | Mortality per cent. | Trade. | Mortality per cent. |
|---|---|---|---|
| House-porters, both sexes | 33 | Stonecutters | 20·75 |
| Ropemakers | 25 | Saddlers | 20·5 |
| Laundresses | 23 | Cooks | 20 |
| Labourers | 21·5 | Sick-nurses | 20 |

* In the plans for workhouses sanctioned by the English Poor Law Commissioners, and published in their first Report to Parliament, only 108 cubic feet of space is allowed to each inmate.

| Trade. | Mortality per cent. | Trade. | Mortality per cent. |
|---|---|---|---|
| Metal-founders | 19·5 | Tailors | 12 |
| Carders, of both sexes | 19 | Painters | 12 |
| Quarrymen | 18 | Thrashers | 12 |
| Bootmakers | 16 | Dressmakers | 11·6 |
| Valets de place, or waiters | 16 | Jewellers | 11·6 |
| Journeymen | 15 | Sawyers | 11 |
| Water-carriers | 15 | Locksmiths | 10·5 |
| Carters | 15 | Printers | 9·5 |
| Gardeners | 14·5 | Bakers | 9·2 |
| Joiners | 14·5 | Frameworkers | 9·1 |
| Butchers | 14·5 | Gilders on Wood | 9 |
| Prostitutes | 13·9 | Paviers | 5·5 |
| Female spinners (?) | 13· | Soldiers | 4·5 |
| Carpenters | 13 | | |

(Table viii. D. p. 566. Tom. ii.)

When discussing the numerous details that come successively under his notice, Dr. Motard adopts a tone of moderation and impartiality in stating facts and drawing inferences; so that, although his arguments may not be always convincing, his method, at least, is quite satisfactory. As an example, we would refer to his examination of the disputed question, whether the emanation from animal matters in a state of putrefaction be injurious or not to the living. Contrary to popular belief and the unanimous opinion of writers on the subject, Parent Du Chatelet in France, and Dr. Warren in the United States, maintained that these emanations were not only innocuous, but actually beneficial in giving immunity from the attacks of epidemic diseases to those within the range of their influence. Dr. Motard briefly relates the facts advanced on both sides, and observes that positive facts are always more weighty than negative; that animal poisons do really produce dreadful results when introduced into the system by inoculation; and as absorption into the system may take place, analogy is in favour of the positive facts. He then discusses the chemistry of the question, and concludes with these inferences: A. There are three kinds of putrefaction; 1, mammificated air or water, having no access to the animal substance, this species is perfectly innocuous; 2, exposure to the air and decomposition into gaseous products and a carbonaceous deposit; odour fœtid; very slightly innoxious; 3, a mixed kind of putrefaction dependent on a limited supply of air, or excited by a fermentative principle itself the product of a limited supply of oxygen; odour variable, but usually disgusting; noxiousness very great. B. In all the facts quoted by Du Chatelet and Warren, the putrefaction had taken place in the open air; in all those on the opposite side, the contrary was the case. C. The varying circumstances under which the exposure to the emanations takes place necessarily lead to varying results. For example, the system may become habituated to a poison applied in moderate doses; or the season of the year, or hour of the day, the temperament of the individual or state of health and susceptibility of absorption will modify the results. D. The virulence of the emanations may be counteracted by a plentiful supply of fresh air, and a frequent renewal of it. We may add that a similar eclectic method is adopted in discussing the contagiousness or non-contagiousness of the plague, yellow fever, &c. and facts apparently conflicting are made to harmonize in a satisfactory manner, both parties in this fierce dispute being right and both wrong.

Dr. Motard draws a comparison between the vital statistics of the past and present century highly favorable to a system of public hygiène. The mortality in Paris has diminished just in proportion as the public health has been protected by hygiènic measures. In the year 1350 the mortality was so high as 1 in 17; in 1660 it was 1 in 25; in 1821, 1 in 32; in 1826, 1 in 36. In 1750 the mortality in London was 1 in 20; and from 1744 to 1800, the deaths out-numbered the births by 267,000; in 1821 the mortality had fallen to 1 in 40; and from 1801 to 1830, the births exceeded the deaths by 102,975. Epidemics are fatal in proportion as the population is crowded [and poor.] This was shown in the official report on the cholera in Paris. The number attacked in the city quarter was thirty times greater and the deaths twenty times more numerous than in the quarter of the Tuileries.

We shall now take up Dr. Motard's essay conjointly with Mr. Alison's, and notice from time to time the opinions and statements in which the two writers agree. It appears that Mr. Alison wrote the first draft of his work more than thirty years ago, when he had just terminated his philosophical studies, and when the doctrines of Malthus were making a great impression. From 1810 to 1819 was occupied in making observations and adding them to the work, which at last became so voluminous that Mr. Alison determined to rewrite it. It was not, however, until 1828 that this task was completed; but when completed, it appeared to the author that the time for successful publication was past; and laying aside the manuscript for his executors, he immediately commenced "The History of Europe during the French Revolution." The process of reaction has been developed sooner than Mr. Alison expected, and has induced him forthwith to launch his principles on the advancing wave of anti-malthusian legislation.

Mr. Malthus lays it down that population increases in a geometrical, food only in an arithmetical progression; that therefore in every country long inhabited, population must necessarily press on the means of subsistence; and that its progress beyond these means is limited only by the *positive* checks of vice and misery, or the *preventive* check of moral restraint. To these principles Mr. Alison opposes his own. He does not deny the power of moral restraints, but on the contrary asserts that they are not merely preventive, but positive checks on population; but he asserts also that they cannot coexist with vice and misery: while vice and misery, so far from being positive checks on population, will of themselves, under certain circumstances, encourage the excessive multiplication of the human race.

"It is a most remarkable fact, totally at variance with what might *à priori* be expected, but confirmed by the universal experience of mankind, that the dominion of reason over the passions, the habit of foresight, and the power of forming a systematic plan for the conduct of life, are just in proportion to *the degree in which the danger of immediate want, or the pressure of actual suffering have been* REMOVED *from mankind*." (Vol. i. p. 243.)

"A certain degree of poverty among the labouring classes totally annihilates all limitations on the principle of increase, and renders early marriages as universal as the indigence which creates them. Men never look before them *unless they have some permanent object to look to ;* they are always influenced exclusively by present enjoyments, unless they see some sufficient reason to forego them." (Vol. ii. p. 43.)

Referring to Dr. Motard's definition of hygiène, it will be seen that he and Mr. Alison have a common object in view, but approach it by the different routes of political economy and medicine, using the latter term in its widest sense, as comprising the science of human physiology and pathology. It is, then, as a contribution to the philosophy of hygiène, and as discussing some, and only some, of those moral wants of man, which Dr. Motard has not specifically considered, that we present "The Principles of Population" to the notice of our readers.

According to Mr. Alison the true moral checks upon population result from the agency of certain desires, which he terms artificial wants. These wants are developed by civilization, and become more powerful in their operation as civilization advances, and when, theoretically, there is the greatest danger of the population increasing beyond the means of subsistence. One of these wants is the want of animal comforts and luxuries; the desire to eat wheaten instead of rye-bread, to drink beer and tea instead of train oil or ditch-water, to wear fine clothes, &c. But "the desire to better one's condition" gives rise to the most powerful moral checks, *we* would add, also to the greatest moral evils. The mechanic feels pride in attaining to the dignity of a name-plate and brass-rapper on his door, his father being a decent man but a step below such grandeur. The wealthy tradesman builds his villa, and his daughters never rest till they get amongst "the carriage people," and not even then. The rich manufacturer aims at lands and a title; and the head of an ancient noble house aspires to be the head of the government, or at least of one of the parties in his country. We all want to rise; and as a decent coat is the first step from the mud of poverty, and indicates the birth of ambition and the first dawnings of rational enjoyment, foresight, and prudence, we may readily agree with Mr. Alison when he asserts that "tailors and milliners would do more in the end to improve the habits of the lower orders than all the efforts of the benevolent." Our readers will at once assent to the correctness of the following illustration:

"It is the continual pressure *from below* which occasions the excessive competition in every profession and business of life. Ask the physician, the lawyer, the tradesman, or the merchant, to what cause the difficulty they experience in making their way in the world is to be ascribed, and they will all answer that it is the influx of persons into their professions from an *inferior rank* which creates the competition." (Vol. i. p. 112.)

The application of this principle is obvious. A young man will take care how he embarrasses himself in his struggle upwards, with the dead weight of a wife and children. If he marry at all, it is not until the age of thirty of forty that he can do so safely, an age at which, according to Quetelet, the fecundity of marriage is diminished one half. The rate of increase is slowest with the most opulent; that is with those who have the most expensive artificial wants, and increases as we descend in the scale of society.

Mr. Alison next proceeds to analyse the circumstances by which in societies of mankind these principles are modified; and developes in an interesting and comprehensive manner the influence which climates, government, laws, religions, and customs have or have had upon human happiness. He passes in review over every country and through every age, including our own, with a special reference to the double effect which the modifying circumstances have in increasing or diminishing the ease and welfare of mankind. The result is, that he shows Malthus to be in error when he asserts that "the misery produced by government is slight and superficial, compared with those deep-rooted seeds of evil which have their origin in the principles of human nature." So far from this being true, it is clear from the history of all times and of all nations, that bad government, unwise laws, and false religions, invariably induce social misery and degradation. Bad government renders property insecure, and therefore industry is relaxed, for wealth cannot be accumulated, and if wealth cannot be accumulated, artificial wants will not be developed, because they cannot be enjoyed. Nor can "the desire of bettering one's condition" exist if gradations in rank be interfered with by *castes* or sumptuary laws; or annihilated by despotism, whether aristocratic or ultra-democratic. Good government and wise laws have invariably contributed to the welfare of man.

Mr. Alison and Dr. Motard are agreed in several points. One we will notice. Of the nations of the north the latter author observes, when discussing the moral influence of climate:

"In the northern latitudes man requires an abundance of food, and as the soil does not supply it, necessity and habit render him carnivorous. He is consequently shepherd and hunter, nomade and sanguinary, warlike, and inured to danger. Such being his character, he despises the peaceable inhabitant of a more fertile soil. The warlike German tribes who left their forests to overrun Europe, and the Scythians who have invaded Asia from the most remote periods, always exhibited these characteristics. But none have shown them in so terrible a manner as those robbers of the Mongolian race, who are accustomed to sweep in their chariots over that immense plain of Northern Asia which extends from China to the Oby, and from the Great Desert to the Icy Ocean. Three times in particular have they terrified the world by their fearful irruptions, massacring and burning all before them; and leaving nothing in their track but the quiet of the desert and the stillness of the tomb. Attila, their first leader, wished that no grass might grow where his horse had trod; and Tamerlane, the most civilized of them all, erected at Bagdad, a horrible trophy of 90,000 human heads. Depopulated Asia still shows traces of their presence." (Motard, tom. i. p. 106.)

It is further shown that the inhabitants of northern climates are the most virtuous, and exhibit most of the dignity of the human character, in accordance with the doctrines of Montesquieu. Morals are more depressed as we approach to the southern latitudes; the passions more violent, crimes more numerous. Dr. Alison thus writes:

"How much soever wealth and luxury may corrupt their possessors, in the rich and fertile districts of the globe, there are always some situations in which the influence of such sources of depravity cannot be generally felt. In the cold and sterile regions of the north, and in the desert plains of the south, wealth cannot be accumulated, and men decay. Scythia and Arabia, accordingly, have in every age been peopled by inhabitants leading the same nomade life, and possessing the same active and hardy character. .......... If the Scythians and Arabs do not share in the improvement of civilization, they are not weakened by its vices; if they are stained by the cruelty of savage manners, they possess the energy of the savage character. It is from these great high-lands of humanity that the stream of conquest has in every age flowed down upon the inferior scenes of existence. It is in their recesses beyond the reach of dissolution, that the energy necessary to sustain the human character is prepared; like the glaciers in the physical world, which the sun of summer is unable to dissolve, and from whence, in the solitude of the Alps, those undecaying fountains are fed which spread life and fertility through the surrounding plains. .. ........ When an empire had risen to eminence in ancient times, its inhabitants became corrupted, and its cities swarmed with a degraded population. But the shepherds of the north were always the same, and in the solitude of their deserts, nature was preparing the regeneration of the world. While the civilized empires of the east were advancing in wealth, in numbers, and in wickedness, the wandering tribes of Scythia were preparing to burst from their deserts in quest of subsistence. Unmarked as it was, amid the blaze of military glory, the fatal hour was approaching which was to witness their fall; and the cloud, which seemed at first only a speck on the horizon, swelled till it had buried the universe in its darkness." (Alison, vol. i. p. 264.)

Taking a practical view of the high questions here started, we should doubt much, as physiologists, whether any system of public hygiène could effectually resist the influence of an enervating climate on man, or modify the thick neck and broad jaws of the Mongol, so indicative of his destructiveness. It seems to us that the customs and habits created by climate induce changes in the cerebral organization of nations, as well as in the muscular and osseous conformation, and that the mental and corporeal qualities which result from these changes becoming hereditary, characterize the race. We know that lower animals when subject to change of climate and habits, or in other words, when domesticated, acquire new instincts contemporaneously with peculiarities in the form of the body; and that distinct breeds of the same species are thus developed. The character of a government is unquestionably regulated all over the world by the character of the people governed; and if the climate determine the character, by stamping its effects on the mental and physical organization of its inhabitants, how can we hope to discover or apply successfully principles of government or of hygiène, not simply powerful enough to resist its ever-acting influences, but able also to change those vices of organization which have been the growth of ages? All experience is against us. The moral character of the French at the present moment is given by Julius Cæsar, when he describes the character of the Gauls; and what says Mr. Alison of the nomade Tartar tribes, of China, with its throne supported by the press, or of India with its ancient civilization?

"The Tartars of the present day differ in no respect from their ancestors in the days of Herodotus; and in the manners of the wandering tribes who now infest the deserts of Mesopotamia, we are transported to the days when Abraham sojourned in the land of Urr." (Vol. i. p. 265.)

"To whatever cause it may be owing, nothing is more certain than that the government and institutions of the oriental states are precisely the same at this time as they were at the earliest period of which history makes mention. The descriptions of Porter, of Buckingham, of Morier, of Fraser, differ in no respect from the picture which may be gathered from the graphic sketches of Herodotus; and the most faithful portrait that ever has been given of the present manners of Bagdad and Ispahan, is that which for a thousand years has given delight to every successive generation, in the Arabian Night's Entertainments." (Ibid. p. 399.)

The physiological doctrines we have advanced would be incorrect, if facts were otherwise; but the melancholy practical inference to be deduced from the theory and the facts is, that it is just as easy to educate a Bengalee into a Mongol, or an Italian greyhound into a Newfoundland dog, as to teach the Hindoo how to enjoy and maintain a free government. Ten centuries would be uselessly spent in the attempt to annul the *climatic* effects of fifty or sixty, perhaps of a hundred.

We do not make these statements with any other wish than to render philanthropists and legislators aware of the important assistance they may derive from a knowledge of medicine, or rather of the absolute necessity they are under to acquire and act upon such knowledge, if they would act aright. And with this object in view, we will mention other considerations arising out of the subject. Great Britain in establishing her colonies is in reality founding empires, which at some future period will be greater than any that have yet appeared in Asia. "There is something solemn," says Mr. Alison, "and almost awful in the incessant advance of the great stream of civilization, which in America is continually rolling down from the summit of the Alleghany mountains, and overspreading the boundless forests of the far west. Nothing similar was ever witnessed in the world before." The plundering multitudes of Scythians and Tartars that poured into Asia are as nothing compared to this torrent. Not less than 300,000 persons, almost all in the prime of life, now yearly cross the Alleghany mountains, and settle on the banks of the Ohio or its tributary streams, and they settle never to return. Should the inhabitants of the United States continue to increase at the same ratio as they increase now, in fifty-eight years they will number 100,000,000 of souls. The basin of the Mississippi alone, if as densely peopled as the British Isles, (it is incomparably more fertile,) would contain upwards of 350,000,000 of inhabitants; but under favorable political circumstances it would easily support more than 1000,000,000. The same process is going on in Australasia and Polynesia. "We behold the British race peopling alike the western and southern hemisphere, and can already anticipate the time when 200,000,000 of men on the shores of the Atlantic and in the Isles of the Pacific, will be speaking our language, reading our authors, glorying in our descent." (Alison, vol. ii. p. 348.) Need we say that the responsibility of British statesmen and of the British nation is most solemn? In two or three centuries a larger population than exists in the whole of Europe will curse or bless us according as we have given a bias for good or evil to their infant institutions. Reverting to our previous remarks we would, as physiologists, warn our colonial secretary and our colonizing companies against the mixture of superior with inferior races of men; the Hill Coolie with the Highlander, the African with the Englishman. Liberty and a real equality are necessarily coexistent; and we should have no difficulty in proving that such a mixture of races will inevitably lead to slavery and despotism, to destructive foreign and civil wars, and a retrograde civilization; and for the primeval solitudes now being broken upon, will substitute the silent ruins of desolated cities.

Climate will undoubtedly change the character of the English race. It changes it in India; it is changing it in the United States, and in less than a century will dissolve the union. It is of importance, then, in marking the limits of new colonies, to consider the ultimate effects of climate, and place natural boundaries between them. When the United States separate, the northern will coalesce with the Canadas, and these unitedly will constitute the dominant empire of the western continent, and perhaps of the world. These changes will hardly take place without wars; and the length and destructiveness of these wars will depend considerably upon the nature of the boundaries, and the compactness of the territories to be defended. Portions of our empire in India might be garrisoned by colonies. The climate of the high lands in central Asia so nearly resembles our own (as do also the inhabitants ourselves), that Englishmen would not deteriorate there; and would do more for the civilization of Asia and the glory of England than innumerable colleges and missionaries in Hindostan. We would close our observations on this part of the subject with the hope that British statesmen and legislators will ere long be able to estimate properly the lives of Englishmen as the lives of men springing from a race in whose cerebral organization the ideas of rational freedom and self-government are stereotyped by climate, laws, and habits, and so have become instinctive; and will adopt those measures best calculated to enhance the value of such a population by lessening the mortality and ameliorating the condition of the labouring poor.

It is with the means best calculated to effect this purpose that the second volume of Mr. Alison's work is principally occupied. In the first chapter the results following the acquisition of landed property by the poor are discussed, as well as the nature of the government, when land is possessed by few or many proprietors. France, during the revolution, uprooted her hereditary aristocracy, and repealed the law of primogeniture. She thus, according to Mr. Alison, created 10,872,000 landed proprietors, whose territorial possessions average eleven acres and a half, and their clear annual profits £4 5*s.*, or half the value of the produce. Mr. Alison thinks that by these two acts, France exchanged European for Asiatic civilization; and asserts that her peasants are fast sinking into the condition of the ryots of Hindostan. But what was the condition of France before the revolution? The despotism of her court was oriental, the sensuality and servility of her nobility were oriental, the oppression of her peasantry was oriental. The fruits of orientalism were ripe when the revolution took place; the gathering of them in fact constituted the revolution. The government of France is oriental still; but not because the land is subdivided. It is the character of the people that determines the character of the government, as we have before observed, a general principle illustrated by the history of France. The extreme division of land has not led to that increased cultivation and increased produce which might have been expected. In Great Britain, according to Dr. Motard, each agriculturist produces annually to the value of 720 francs, or £28 16*s.*; in France it is only 234 francs, or about £9 7*s.* (vol. ii. p. 239.) And why? The French *peasantry*, whether in France or Lower Canada, are oriental in their nature; they are "not given to change."

The eleventh chapter is on the moral evils and management of the poor in great cities; a subject peculiarly within the scope of hygiène, even in its more limited sense. We have a fearful description of urban populations, especially of our own, and of the evils which will inevitably result if we continue to neglect all means of ameliorating their condition. In Paris *every third child is a bastard*, and *one sixth* of the whole population die in the hospitals; in London one tenth of the whole population are paupers, and 20,000 persons rise every morning without knowing where they are to sleep at night; at Glasgow nearly 30,000 persons are every Saturday night in a state of brutal intoxication, and *every twelfth house* is devoted to the sale of spirits; and in Dublin 60,000 persons in *one year* passed through the fever hospital. These are undeniable facts. Mr. Alison testifies to the correctness of the following account of the wynds in Glasgow given by Mr. Symonds, the government commissioner for examining into the condition of the hand-loom weavers:

" 'The wynds in Glasgow comprise a fluctuating population of 15,000 to 30,000 persons. This quarter consists of a labyrinth of lanes, out of which numberless entrances lead into small courts, each with a dunghill reeking in the centre. Revolting as was the outward appearance of these places, I was little prepared for the filth and destitution within. In some of these lodging-rooms (visited at night), we found a whole lair of human beings littered on the floor, sometimes fifteen and twenty, some clothed and some naked; men, women, and children, huddled promiscuously together. Their bed consisted of musty straw intermixed with rags. There was generally little or no furniture in these places; the sole article of comfort was a fire. Thieving and prostitution constitute the main sources of the revenue of this population. No pains seems to be taken to purge this Augean pandemonium, this nucleus of crime, filth, and pestilence, in the centre of the second city in the empire. . . . . A very extensive inspection of the lowest districts of other places, both here and on the continent, never presented anything one-half so bad, either in intensity of pestilence, physical and moral, or in extent proportioned to the population.' (Arts and Artisans at Home and Abroad. By J. C. Symonds, Esq., p. 116, et seq.)" (Alison, vol. ii. p. 89, note.)

Of course in such a population fever slays its thousands. The mortality in the Niger expedition raised a dreadful hubbub, and the public was frantic when it heard of the Cabul massacre. But how little has been said about the wholesale destruction of our countrymen at home! The average annual mortality over all England is 1 in 51; it was 1 in 41 in Glasgow in 1822; in 1835 it had risen to 1 in 29·53; in 1836 to 1 in 26·68; in 1837 to 1 in 24·20. Advocating the necessity for a legal provision for the poor in Scotland, Mr. Alison makes observations which are quite applicable to a system of public hygiène.

"Of the dreadful danger of such a state of things, and the manner in which it speedily comes to affect the higher orders in their lives and property, if they cannot be reached through any other and more honorable channel, decisive proof is afforded by the facts that no less than *twenty thousand* persons were seized with typhus fever, the well-known attendant on want and misery in Glasgow, in the single year 1837, of whom 2180 died; that 40,000 persons have had fever in that city within the last three years; that 10,000 persons have had fever in Dundee in the last four years; that in 1838, 1 in 30 in Edinburgh was a fever patient." (Vol. ii. p. 219.)

Dr. Motard gives similar statistical details; but statistical details of this kind are as dry as they are horrible, so we will exchange them for the opinions of the Bishop of Strasburg. Dr. Motard quotes these from an article which appeared in the "Constitutionnel" for July 15th, 1840. It may be a melancholy satisfaction to know that we are not the only sinners against the labouring poor:

"I have surveyed," says this prelate, "from every point of view one of the departments most celebrated for the grandeur and prosperity of its manufactures, and I could only mourn over the sanatory and moral condition of that country. I shuddered to learn that in these centres of industry the youth of both sexes abandoned themselves to unrestrained licentiousness, and that, in consequence, a population formerly healthy and handsome was deteriorating in the most alarming manner. In addition, the constitutions of the working people are debilitated by their sedentary life and the close foul air of their workshops. I saw poor children going in the evening to these palaces of industry to work all night and earn a few pence, the miserable return for their injured health and wasted youth. The complexion of these victims of avarice was pale, their cheeks hollow, their countenances haggard and deformed. The unhappy children marched with a weary step to their place of torture. In fact, the physical deterioration in some of these establishments peopled by from three to four thousand operatives is so great, and the exemptions from the conscription so numerous in consequence, that a superior recruiting officer loudly declared if the general government did not promptly interfere to remedy the evil, the supply of soldiers from that department would fail altogether." (Motard, t. ii. p. 427.)

It is added from the same article, that at Mulhouse (Mulhausen), formerly celebrated for a fine race of men, the deterioration was so great that for every hundred conscripts found fit for military service, 100 are rejected; but at Rouen 166 (we cannot help giving the figures), and at Elbœuf 168 are rejected for every hundred passed.

Dr. Motard accuses Birmingham of having first introduced the system of infant labour, by commencing a traffic in children with London. This trade, he says, was carried on with such cool impudence that an agreement was entered into between a parish in London and a Lancashire manufacturer, in which it was stipulated that the latter should take one idiot for every twenty healthy children sent to him! The French manufacturers were afterwards compelled to adopt infant labour in self-defence, and now employ more than 100,000 children in their cotton factories alone. (Tom. ii. p. 373.) Mr. Alison apprehends the greatest danger from this system of employing children in factories; he does not appear to have been aware of the horrors of coal mines.

"It may be affirmed, without hesitation, that the system of employing children in these great establishments is the most ruinous to the moral character and habits of increase in a nation that human ingenuity ever devised; and that, if either experience does not discover some remedy for these evils, or Nature, in some way to us inscrutable, does not work out its own cure, the empire will in the end be overturned by their effect." (Vol. i. p. 531.)

"The constant employment of the young in manufactories for fourteen hours a day renders it almost impossible for any education to do them much good; for who, after such a period of daily toil, could sit down to the additional labour of learning anything. It is almost barbarity to propose it............. Nothing but the strong hand of government, and an assessment reaching the vast funds of the selfish and indifferent as well as the humane, is adequate to the remedy of the evil. Whether such a task will ever be undertaken by the legislature, or submitted to by the country, may be doubted; but this may be affirmed, without hesitation, that if this great duty is not discharged, and that, too, without delay, by the nation, the seeds of ruin are, by the laws of God, sown—and justly sown—in the community; and that such will be the depravity and wretchedness of the people on whom the visitation will fall, that even Timour, with his pyramids of ninety-thousand heads, would be deemed a messenger of mercy to mankind." (Ibid. p. 534.)

A factory commissioner, Mr. Tufnell, states in his report that the children that stand at the mules for twelve hours every day have little or nothing to do; and hints that it is quite practicable for them to indulge in literary pursuits! But Dr. Motard, in describing the external aspect of Manchester, "de l'orgueilleuse Manchester," and its population, describes the results of infant labour. We transcribe his pen-and-ink sketch of this manufacturing metropolis:

"As you approach its atmosphere the elegant appearance peculiar to the English villages disappears, and in the suburbs noisy pot-houses take the place of the charming, quiet cottages. What road is that? Why those lugubrious, dull sounds—those blackened walls—that smoky atmosphere? What hell have we dropped into? Everywhere there is the livery of wretchedness, everywhere the degradation of vice: rags—livid, sinister-looking countenances—hoarse voices, mingling the accents of misery with the shouts of brutality. Men aged before they are old, rickety children, women no longer like their sex; everywhere an etiolated population, everywhere furious groups demanding bread. From whence come those vociferations? It is the riot of the famished, the most implacable of all, the prelude to pillage, and robbery, and incendiarism. Oh, Manchester! in whose palaces of industry which human genius has created within thy walls, how often has hideous famine polluted with its rags thy brilliant wonders! How often hast thou seen thy thousands of operatives seize with seditious hand the bread of the rich! In fact," &c.

And then Dr. Motard clinches his poetry with the prosaic dates of riots, sabreings, and soup-kitchens.

Whatever may be the cause, it is quite certain that extreme poverty and incredible misery are found in large manufacturing populations. Mr. Alison connects these evils with the unequal distribution of wealth, and mentions Ireland, an agricultural country, in illustration. Dr. Motard shows numerically that pauperism increases not in the ratio of the population, but in the ratio of the commercial and manufacturing prosperity. In ten years (from 1790 to 1800), the population increased at Philadelphia 29 per cent., the paupers 104 per cent.; from 1800 to 1809 the latter increased 79 per cent., the population only 36 per cent. (Tom. ii. p. 377.) But Glasgow itself is a much more striking example of this general fact.

The remedies for these evils are of a mixed character. Mr. Alison sees in the constant improvements in machinery a providential check upon the increase of the manufacturing masses and a stimulus to increased agricultural occupation. Education, but particularly a religious education, emigration, and a legal provision for the poor are the principal means he thinks necessary to be adopted. He also discusses the corn laws and the reciprocity system, but with these we have nothing to do. A system of medical police is one of his minor measures for the improvement of the population in large cities; and he would have it enforced by law.

"It is in vain to say that this matter (the building of streets) may, without danger, be left to the interest of individuals, and that every proprietor should be allowed to lay out his property to the best advantage. The interests of the higher and middling orders, indeed, may be safely intrusted to their own keeping; but it is neither just to the poor nor expedient for the public, to leave the indigent classes at the mercy of their superiors.............. It is in protecting extreme indigence from the necessities to which it would otherwise be compelled to submit, and in enforcing police regulations—important alike to the health, the manners, and the morals of the lower orders—that the power of government is most beneficially exerted. Like the laws of quarantine or of public cleanliness, such regulations are necessary to enforce those salutary rules, which avarice is always ready to violate, and indigence too often unwilling to obey." (Vol. ii. p. 127 et seq.)

Mr. Alison founds an argument in support of a legal provision for the poor upon the general principle that mendicity increases where property is unequally distributed. We may add that Dr. Motard has similar views, but not being clearly worked out, they are not clearly expressed. This argument of Mr. Alison's being equally powerful in asserting the justice of a system of public hygiène, we are induced to quote it, premising, that it is not a favorable specimen of Mr. Alison's style:

"The accumulation of the labouring classes in great masses in the employment of their superiors is eminently favorable to the increase of wealth. The profits made by master-manufacturers or merchants who have numbers of the poor in their employment are prodigious............... All the other classes share in the advantages of the wealth which is thus created:—the agriculturists in the increased market for their produce which is thus created—the tradesmen and artificers in the augmented demand for their productions to which it gives rise. The operative workmen, perhaps, share least in the advantages of the wealth which their industry has created: degraded and demoralized by the habits which it induces, they are almost precluded from acquiring any permanent benefits from the skill which they possess. In these circumstances, the security of a provision in sickness or old age is nothing more than a fair compensation for the risks which they run and the contamination to which they are exposed: for health which is undermined, morals which are corrupted, foresight which is destroyed, sensuality which is disseminated. To resist such evils is as impossible, in certain circumstances, as for soldiers to preserve their health amidst the dysenteries of the camp, or the contagion of the hospital: to relieve them is the duty equally of a wise general and beneficent government." (Vol. ii. p. 186.)

The operative is in fact a serf (to speak plainly), without the protection of a master. He is not *adstrictus glebæ*, but he is bound by the iron band of his necessities to a course of life fraught with the evils Mr. Alison so correctly details. His employer, unlike the feudal baron, has no immediate interest in his welfare, is not directly dependent upon his arm for power, for protection, or for the necessaries of life. Labour is so abundant that he is virtually a slave, yet without the animal comforts of a slave; his necessities constituting a most efficient substitute for the driver's whip and collar. Education opens his eyes only to show him that he is irremediably naked; it gives him the desire to better his condition without the means; and what, we ask, will men, when goaded on by pride, rancorous envy, vicious sensuality, and the desperation of intolerable poverty, hesitate to do?

If we do not at once express our concurrence with the gloomy views of Dr. Motard and Mr. Alison, it is because they have altogether neglected to consider a most important, perhaps the most important product of modern civilization. For the first time in the history of mankind the medical profession stands alone, uncontrolled by the monarch or the priest. Hitherto it has never been powerful but as the servant of tyranny and superstition; now it depends on itself only whether it shall serve the cause of justice, mercy, and truth. Its individual members cultivate science for the benefit of humanity and to alleviate human misery in its detail; but the necessities of the age imperatively demand that they shall act as a body in the state, that collectively they shall place themselves in the same relations to society as, individually, they bear to their individual patients. The united profession must insist that its reasonable demand to be intrusted with the execution of plans for securing the health and ease of the community be conceded. To be the appointed instruments of carrying out a system of general hygiène is its right; to constitute the machinery by which natural science may be applied to the amelioration of the evils of society is its duty. The natural sciences have been mainly developed by the profession; and, if natural science in aiding manufacturing industry has indirectly created a great amount of human misery, the profession which has nurtured it to its present pitch of power must apply that power to the removal of its own baneful results. We agree with Dr. Motard when he asserts that it is medicine alone which can untie the gordian knots of modern civilization. But medicine must be *applied* to society in all its ramifications; and it is the medical profession that ought to, indeed that only can apply it. It is the medical profession which can most effectually step in between capital and labour; and not simply show, theoretically, how the condition of the labourer may be improved, but so guide and control public opinion, that avarice itself shall be made subservient to that improvement. The modern slave may then possibly be placed in that position in which it will be well for himself and well for society; and a servile war in Europe, the most terrible blow that could be struck at the present or future happiness of mankind, would be prevented. We believe the political institutions of all other nations are such that this arduous duty will devolve upon the medical profession of the British empire. Religion apart, we can imagine no greater or nobler enterprise in which a body of educated men could engage than this. The concentration of moral and physical science on the physical and moral advancement of mankind must, confessedly, be a great object for even such a body of men to aim at; but it is a greater to become the rallying point and centre of action for that active and zealous philanthropy which happily grows with the evils of the age, and wants but an enlightened guidance to be equal to the task of counteracting them.

Self-sufficient shrewd people may laugh and say "there's nothing like leather;" but we appeal to the medical profession, and ask them if the relations they bear to society are not of surpassing importance. The study of modern medicine is the study of human nature, and being such there are no questions concerning humanity which it may not assist to solve. This may be generally inferred from our sketch of Dr. Motard's essay, and we have given a solitary instance of its application to one of the most profound political questions of modern times, namely, the founding of colonies. Our mission enterprises, diplomacy, and commerce have been admirably served by medical practitioners, and might be to a much greater extent. Forensic medicine, especially in its relations to the doctrines of free-will and moral responsibility, is as yet scarcely developed. Eleemosynary medicine or the relief of the sick poor is equally imperfect; and so is public hygiène, whether we consider the application of it to the arrest of epidemics, the purification of the air of cities, the construction of streets and public buildings, or the management of manufactories, workhouses, prisons, &c. Our quarantine laws are ridiculously absurd. No systematic attempt has been made to foresee bad harvests or the recurrence of epidemics, and so to ameliorate these unavoidable evils by anticipating them. We have no medical observatories, yet this good is within the scope of medicine. We need not state how vast is the importance of a right system of medical education if the duties and power of the profession be such as we have stated. These are only a few and the most obvious of the important relations which medicine bears to society.

In giving a general estimate of the works before us, we would observe that the error in style so apparent in Dr. Virey's is also apparent in Mr. Alison's essay, and we may even add, in Dr. Motard's. The object of hygiène is a great and good object; it is in fact the great object of Christianity, and therefore is essentially noble and godlike. Writers on hygiène feeling this, are easily betrayed into a corresponding grandeur of diction which appears ridiculous to those whose thoughts have not been occupied with the subject. In short it looks like cant, and so tends to retard the practical application of the sciences. Great truths are most touching and most attractive when expressed in simple language; besides, writers who declaim much are apt to think when they have done that they have done enough. But that is not a sound opinion. "*Sola laus virtutis* in actione *consistit.*" We must *act*, and act on a scale, and with an energy and a determination proportionate to the greatness of the object to be attained.

Dr. Alison's principal defect is diffuseness. His "Principles" are valuable; and we shall be happy if any remarks of ours induce the profession to read his work, for we think that they will learn from the perusal to appreciate their own importance and usefulness to the community. If the whole were condensed into a half-guinea volume (an easy task), it would be more within the reach of many of our readers.

Dr. Motard's essay exhibits all the comprehensiveness and system which so eminently characterize French literature in general, besides much of that practical merit we claim as peculiar to our own. It would almost bear translation. The book is imperfect; but it is imperfect mainly because it occupies only two octavo volumes widely printed. In the present state of the science general hygiène cannot be thoroughly discussed in two such volumes; it is too rudimentary and requires too many details.

We shall take an early opportunity to discuss our own system of public hygiène, and notice especially the reports which within the last few years have been made to parliament by commissioners appointed to inquire into the health of towns and the condition of the manufacturing and mining populations. We shall examine their contents and discuss the relations they bear to the medical profession,—to *physicians* in the true meaning of the term, and not to mere curers of aches and ailments. We indulge a faint hope that in discussing the questions connected with general hygiène, we shall convince Mr. Whewell that medicine is actually a science after all he has said to the contrary.

